# Bioactive Molecules Released in Food by Lactic Acid Bacteria: Encrypted Peptides and Biogenic Amines

**DOI:** 10.3389/fmicb.2016.00876

**Published:** 2016-06-09

**Authors:** Enrica Pessione, Simona Cirrincione

**Affiliations:** Laboratory of Biochemistry, Proteomics and Metabolic Engineering of Prokaryotes, Department of Life Sciences and Systems Biology, University of TorinoTorino, Italy

**Keywords:** opioid, antioxidant, chaperon-like, cell-cycle control, antimicrobial peptides, beta-phenylethylamine, tyramine

## Abstract

Lactic acid bacteria (LAB) can produce a huge amount of bioactive compounds. Since their elective habitat is food, especially dairy but also vegetal food, it is frequent to find bioactive molecules in fermented products. Sometimes these compounds can have adverse effects on human health such as biogenic amines (tyramine and histamine), causing allergies, hypertensive crises, and headache. However, some LAB products also display benefits for the consumers. In the present review article, the main nitrogen compounds produced by LAB are considered. Besides biogenic amines derived from the amino acids tyrosine, histidine, phenylalanine, lysine, ornithine, and glutamate by decarboxylation, interesting peptides can be decrypted by the proteolytic activity of LAB. LAB proteolytic system is very efficient in releasing encrypted molecules from several proteins present in different food matrices. Alpha and beta-caseins, albumin and globulin from milk and dairy products, rubisco from spinach, beta-conglycinin from soy and gluten from cereals constitute a good source of important bioactive compounds. These encrypted peptides are able to control nutrition (mineral absorption and oxidative stress protection), metabolism (blood glucose and cholesterol lowering) cardiovascular function (antithrombotic and hypotensive action), infection (microbial inhibition and immunomodulation) and gut-brain axis (opioids and anti-opioids controlling mood and food intake). Very recent results underline the role of food-encrypted peptides in protein folding (chaperone-like molecules) as well as in cell cycle and apoptosis control, suggesting new and positive aspects of fermented food, still unexplored. In this context, the detailed (transcriptomic, proteomic, and metabolomic) characterization of LAB of food interest (as starters, biocontrol agents, nutraceuticals, and probiotics) can supply a solid evidence-based science to support beneficial effects and it is a promising approach as well to obtain functional food. The detailed knowledge of the modulation of human physiology, exploiting the health-promoting properties of fermented food, is an open field of investigation that will constitute the next challenge.

## Introduction

Human health is the result of a correct physiological status often resulting from the reciprocal interaction of gene-derived signals (genetics) and environment–generated information (epigenetics). Recognizing gene signals is relatively easy whereas environmental stimuli are often multiple, complex, and reciprocally interacting. Temperature, pH, redox balance, sleep, diet, drugs, and psychological status can deeply affect gene expression, metabolic pathways, and homeostasis (Carey, 2012). However, human genes and environment are not the only players: microorganisms can take part to the complex molecular cross-talk existing between external world and “self.”

Firstly, endogenous symbiont microorganisms, the so-called microbiota, can modulate gene expression, induce preferential food intake (Aydin, 2007), influence pH, redox balance and the ratio between pro-inflammatory and anti-inflammatory cytokines (Belkaid and Hand, 2014). Briefly, control brain, metabolism, immune system, and several homeostatic routes.

Secondarily, food-derived bacteria and yeasts can exogenously affect nutritional parameters, metabolism, oxidative status, immunity, blood pressure, appetite, behavior, also controlling the endogenous microbiota (Arora et al., 2013) by altering the ratio among saccharolytic and proteolytic species, by modulating symbionts gene expression and several other functions (O’Flaerty, 2014). Hence, all fermented food, containing living organisms, can contribute to modulation of the host physiological balance and it constitutes an opportunity to enrich the diet with new bioactive molecules finally resulting in phenotypic effects (appetite, cholesterol and blood pressure lowering, improvement of mood, antioxidant, and immune defenses) on humans (Pessione, 2010). Actually, all types of modulations occur *via* a complex network of signals, among which proteinaceous compounds play a crucial role.

Microorganisms are able to synthesize a large number of metabolites with assessed beneficial or detrimental properties for human health. Among these, nitrogen-bearing molecules such as amino acids, amino acid derivatives and oligopeptides have received great attention since they can affect human physiology in multiple ways.

As an example, amino acid derivatives such as selenocysteines and selenomethionines, have recently been reported to be biosynthesized in both Lactobacilli (Lamberti et al., 2011) and yeasts (Porto et al., 2015). Although selenoaminoacids are not true “bioactive compounds,” directly stimulating receptors on human cells, they can trigger effects deeply affecting human health. The bioactive role of seleno-fixing microorganisms lies in the fact that diet-derived inorganic selenium is toxic (selenate and selenite) or poorly active (elemental selenium) whereas fixed selenium forms (selenomethionines and selenocysteines) are the only bioavailable for humans. On the other hand, only bacteria and yeasts can produce seleno-amino acids from inorganic selenium. Once properly inserted into selenoproteins (i.e., glutathione peroxidase), they can counteract oxidative stress. Besides this well-known antioxidant function, there are data indicating that selenoproteins can modulate immune system (Huang et al., 2012) and activating anabolic circuits such as thyroid hormone biosynthesis (Mullur et al., 2014). Furthermore, epidemiological studies show an inverse correlation between selenium level in blood and cancer mortality, and laboratory experiments have shown a selenium protective effect against cancer initiation and development (Gromadzińska et al., 2008). In *Lactobacillus reuteri* exoproteome studies have demonstrated that two secreted proteins (GAPDH and Phosphoketolase) contain selenocysteines opening the way to employ this strain to supply organic bioavailable forms of selenium (Galano et al., 2013; Mangiapane et al., 2014a,b).

Among amino acid derivatives found in food, biogenic amines are worth of a special mention. Such compounds, although sometimes naturally present (especially in vegetal food) are often the result of the bacterial decarboxylative activity on free amino acids in food. Biogenic amines can be present in non-fermented food, like fish, due to spoilage bacteria that during protein putrefaction release free amino acids undergoing decarboxylation. *E. coli* can produce cadaverine from lysine and putrescine from ornithine (Applebaum et al., 1975). Proteus can produce putrescine from ornithine as a communication signal (Visick and Fuqua, 2005). However, also not-spoiled food, such as fermented food, can present the risk of biogenic amine accumulation. Although starters, exogenously added to perform controlled fermentations, are accurately typed to avoid any risk, autochthonous or contaminant lactic acid bacteria (LAB) can contribute to amine release. LAB are strong amine producers since they use this metabolic pathway (at the place of respiration) to both create a proton gradient and hence energy (for exhaustive review, see Pessione et al., 2010) and to alkalinize the environment, very acidic since their main fermentation products are acids (lactic acid for homofermenter LAB and lactic + formic + acetic acid in heterofermenters).

Many experimental evidences demonstrate that some LAB strains also produce anti-hypertensive, anti-thrombotic, cholesterol-lowering, metal-chelating, antimicrobial, anti-oxidant, immune-modulating, chaperone-like and opioid/opioid antagonist peptides from food proteins (Pessione, 2012), and they can modulate the concentration of opioid and cannabinoid receptors in the gut epithelium (Hayes et al., 2007b). The next sections will focus on some of the referred compounds and will illustrate the main effects exerted on human health.

## Encrypted Bioactive Peptides

Several bioactive peptides, lacking activity when protein-encrypted but acquiring their biological effects when proteolytically released, have been shown to have health-promoting properties as anti-microbials, hypocholesterolemics, opioid and opioid antagonists, angiotensin-converting enzyme inhibitors, anti-thrombotics, immunomodulators, cytomodulators, and anti-oxidants (Hayes et al., 2007b).

**FIGURE 1:**
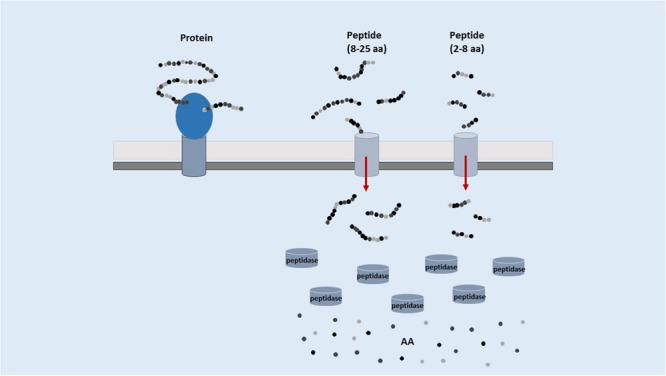
**Schematic representation of the proteolytic system of LAB**.

The human pool of digestive proteases and peptidases can liberate food-encrypted bioactive peptides that can be absorbed by the gut and then reach peripheral organs. However, the enzymatic activity of LAB largely contribute to their release, either into the food matrix (starter or autochthonous LAB) or in the gut (endogenous microbiota or probiotics). LAB are ancient organisms adapted to an anoxic environment that never evolved the capability to biosynthesize heme and hence to have functional cytochromes, catalases, and peroxidases. They are very sensitive to oxygen and devote most of their genes to oxygen stress counteracting. Due to the limited length of the overall genome, the biosynthetic abilities of LAB are very limited especially in amino acid synthesis (Pessione, 2012). Therefore, LAB evolved a complex and sophisticated proteolytic system allowing them to get amino acids from the proteins present in the external environment. A schematic representation of this system, which includes proteases, peptidases and membrane transporters, is referred in **Figure 1**.

### LAB Proteolytic System

Extracellular protein hydrolysis into various long oligopeptides is initiated by a cell-envelope proteinase (CEP). Oligopeptides generated by this first cleavage are subsequently taken up by cells *via* specific transport systems and they undergo further degradation into shorter peptides (bioactive or possible precursors of bioactive compounds) and amino acids in the cytoplasm (Savijoki et al., 2006). In LAB the oligopeptide transporter system (Opp) is the main transporter, belonging to a superfamily of highly conserved ATP-binding cassette transporters (Doeven et al., 2005). The Opp system of *L. lactis* transports peptides up to at least 18 residues (Savijoki et al., 2006). It should be underlined that most bioactive peptides are released into the external environment only when cells undergo autolysis, because only occasionally the longer peptides (originated by the first hydrolytic step) possess biological activity (Meisel and Bockelmann, 1999). Nevertheless, some authors reported that CEP from *Lactobacillus delbrueckii* subsp. *lactis* are suitable to liberate both metal-chelating and anti-hypertensive peptides directly from caseins and without necessity of prior cell autolysis. Maximum CEP activity was a little bit decreased by the addition of NaCl (1%) and glycerol (5%; Hébert et al., 2008). However, proteolytic enzymes released by LAB proved to be very different in the different LAB species and strains, giving rise to a different pool of bioactive peptides (Hébert et al., 1999).

### Food Matrices Containing Encrypted Peptides

Lactic acid bacteria proteolytic system is suitable to produce bioactive peptides from several food proteins, especially caseins that constitute the main nitrogen substrate present in their habitat (milk and milk derivatives; Clare and Swaisgood, 2000). Casein consists of four main proteins (whose ratio is about 38:11:38:13): alpha s1 casein, alpha s2 casein, beta-casein and k-casein, differing in amino acid sequence, hydropathicity index, glycosylation and phosphorylation degree (Pessione, 2012). Usually, CEPs have a strong preference for hydrophobic caseins and *Lactococcus* CEPs have been classified into several types and subtypes depending on their substrate specificity for αS1-, β-, and κ-caseins (Kunji et al., 1996). Casein hydrolysis can give rise mainly to opioid/anti-opioid peptides, antithrombotic and antihypertensive, immunomodulatory, mineral-binding and antimicrobial peptides (Thakur et al., 2012) easily detectable by means of chromatographic techniques after microbial food digestion (Saraswat et al., 2012).

Hydrolytic cleavage of milk whey proteins (alpha-lacto-albumine, beta-lacto-globulin, lactoferrin, and immunoglobulins) can also generate bioactive molecules, such as hypocholesterolemic peptides, which can decrease absorption and enhance fecal extrusion of cholesterol. Actually, milk whey proteins display a greater cholesterol-lowering activity than casein, in particular beta-lactoglobulin (Nagaoka et al., 2001). Beta-lactoglobulin-released peptides also exhibit antioxidant (Hernández-Ledesma et al., 2005) and immune-modulating (Prioult et al., 2004) activity.

Bio-active molecules, especially ACE-inhibiting peptides, can also be found encrypted in bovine meat proteins, such as hemoglobin (i.e., hemorphins) and serum albumin (i.e., serorphin), collagen, elastine, fibrinogen (Minkiewicz et al., 2011; Lafarga et al., 2015).

However, also food of vegetal origin can be a source of bioactive peptides. In soy β-conglycinin β-subunit (Kaneko et al., 2010) and in the large subunit of spinach rubisco, both antihypertensive (Yang Y. et al., 2003) and opioid peptides (i.e., soymorphins-5,-6, and -7, and rubiscolin-5 and -6, respectively), displaying anxiolytic effects, food intake controlling action and enhancement of memory, have been described (Yang et al., 2001; Yang S. et al., 2003; Hirata et al., 2007).

A special mention needs gluten: opioid peptides (i.e., gluten exorphins A4, A5, B4, B5, and C), have been detected and characterized in wheat gluten since 1992 (Fukudome and Yoshikawa, 1992). Some of these peptides have analgesic action on the CNS (Takahashi et al., 2000) but some can also act on opioid receptors located outside the blood–brain barrier, inducing prolactin secretion (Fanciulli et al., 2005). Both antihypertensive (Rizzello et al., 2008) and antioxidant (Coda et al., 2012) peptides are the result of LAB activity on wheat and other cereals. Generally, the high proline content of gluten alpha gliadins prevent hydrolysis by enzymes of the gastrointestinal tract, whereas LAB possess proline-specific peptidase systems. Alternatively, peptide degradation is achieved by the combined action of peptidase from different LAB strains simultaneously present in fermented food (Gerez et al., 2008).

To date, LAB strains able to release bioactive peptides from food proteins, with particular reference to milk caseins, include *L. helveticus* CP790, *L. rhamnosus* GG, *L. bulgaricus* SS1, and *L. lactis* subsp. *cremoris* FT4 (Gobbetti et al., 2002). The hydrolytic ability is related to both the protein substrate, i.e., its amino acid sequence and the proteolytic enzyme panel of each microbial strain (Griffiths and Tellez, 2013). It is possible to enhance (100-fold) the proteolytic potential of LAB by growing them in milk (Wakai and Yamamoto, 2012). Actually it has been demonstrated that peptide-rich media like MRS supplying a ready-to-eat nitrogen source are unfavorable to the induction of most proteolytic enzymes for instance those encoded by Pep N and PepX (Smeianov et al., 2007). On the other hand, CEP are able to hydrolyze caseins only after growth on skim milk but not after MRS pre-culture (Jensen et al., 2009). Generally, lactobacilli genome encodes a larger number of proteases, peptidases, amino acid permeases, and Opp transport systems than lactococci (Klaenhammer et al., 2005). However, more studies on a larger number of species and strains are necessary to establish the true potential of each strain.

Finally, it is worth considering that most information on LAB proteolytic system has been referred to food matrix isolated microflora and to its action on bovine milk casein (Korhonen and Pihlanto, 2003). However, endogenous or probiotic or food –derived LAB can also produce bioactive peptides from the different protein pool present in the host gut, opening interesting perspectives for improving human health (Sharon et al., 2014). In the following paragraphs, the best known bioactive molecules generated from LAB proteases and peptidases are described.

### Antimicrobial Peptides

Lactic acid bacteria proteolytic activity on caseins gives rise to small peptides displaying antimicrobial (both bactericidal and bacteriostatic) activity. Alpha s1 casein and K-casein hydrolysis gives rise to isracidin and k-casecidin, respectively (McCann et al., 2006). Both peptides display inhibitory action on *S. aureus* growth (Meisel and Bockelmann, 1999; Atanasova et al., 2014). K-casein can also originate kappacin, a long bioactive peptide active against *Streptococcus mutans*. This peptide, although deriving from K-casein, is phosphorylated but not glycosylated, and displays the ability to prevent bacterial adhesion to gingival mucosa and to bind enterotoxins (Malkoski et al., 2001). The alpha-s-2 casein fragment 165-203, called casocidin-I can inhibit both *S. carnosus* and *E. coli* (Zucht et al., 1995). *Lactobacillus helveticus* can hydrolyze beta casein, by means of a PR4 proteinase, decrypting an antimicrobial peptide active toward several strains of Gram-positive bacteria (including *S. aureus* and *Listeria innocua*) but also against Gram-negative pathogens such as *E. coli, Salmonella, Yersinia*, (Minervini et al., 2003). Homology studies revealed that this peptide has a similar length to isracidin, but a stronger hydrophobic nature (lacking positive charges) allowing a better activity on Gram-negative bacteria. Furthermore, it bears some proline residues near the C-terminal, which can prolong its half-life rendering this molecule more resistant to the peptidolytic action (Gobbetti et al., 2004). The critical question concerning this peptide is that it has been decrypted from human milk but not from other caseins present in food (cow, buffalo, goat, ship).

Different peptides produced from caseins by different *Lactobacillus* species (*L. acidophilus, L. helveticus, L. plantarum, and L. rhamnosus*) and by *Lactococcus lactis* are active against Gram-negative rods such as *Enterobacter sakazakii* (Hayes et al., 2006). An interesting antimicrobial peptide, named Lactoferricin B, having broad spectrum of action including Gram-negative and Gram-positive bacteria, yeasts and filamentous fungi, has been described as the result of the hydrolytic action on bovine lactoferrin (Dionysius and Milne, 1997; Kitts and Weiler, 2003). This cationic peptide alters membrane permeability with consequent dissipation of the proton gradient and has therefore bactericidal action (FitzGerald and Murray, 2006). It was cloned and expressed in *E. coli*, showing activity against *Klebsiella pneumoniae, Streptococcus Mutans*, and *S. aureus* (Luo et al., 2007). Lactoferricin B is especially active toward the enterohemorrhagic strain of *E. coli* 0157h:7 (Muro-Urista et al., 2011) and proved to be active also against wine-spoilage LAB such as *Oenococcus oeni, Pediococcus damnosus*, and *Lactobacillus brevis* (Enrique et al., 2009). It is difficult to establish the real LAB potential in hydrolyzing lactoferrin. Some experiments were performed by using both microbial-derived enzymes and fermentation with proteolytic starter cultures (Sharma et al., 2011), however, the true potential of each strain has to be experimentally proved since a great variability was observed among strains (Korhonen, 2009). More recently, the proteolytic activity of different LAB strains on goat milk beta casein and beta-lactoglobulin were tested. *Lactococcus lactis l598, Lactobacillus lactis 1043, Streptococcus thermophilus t3D1, Dt1* and *Lb. delbrueckii* subsp. *bulgaricus b38, b122* and *b24* revealed a significant potential in producing antimicrobial peptides inhibiting *S. aureus, L. monocytogenes, Listeria innocua, Enterobacter aerogenes*, and *Salmonella enteritidis* (Atanasova et al., 2014).

Concerning the mechanism of action of such antibacterial compounds, several models have been proposed. Being amphipathic molecules, bearing a hydrophobic moiety and a strongly positively charged domain, this peptides can firstly bind to teichoic acids in Gram-positive-, or to LPS in Gram-negative bacteria (Vorland, 1999) and then interact with the negatively charged bacterial membranes. Most of them act as proton gradient perturbing molecules, (causing membrane depolarization like polymyxin B and colistin; Lohner and Blondelle, 2005), however, some authors suggest that also removal/chelating of membrane-bound iron can affect bacterial viability (Sharma et al., 2011). For what concern lactoferrin-derived peptides, however, it has long been established (Bellamy et al., 1992) that the domains of lactoferrin involved in the antibacterial activity are different from those involved in iron binding. Complex mechanism, including inhibition of the synthesis of macromolecules (Ulvatne et al., 2004) as well as synergic action with host innate immunity compounds were also reported (Brogden, 2005).

These antimicrobial compounds are appreciated in the food industry (especially dairy industry) as natural preservatives counteracting undesired contamination. This allows to reduce the amount of sugar and salt with excellent benefits for diabetics, obese, and hypertensive subjects. Furthermore, their stability in blood and serum render them promising infection control agents. Some of them are also good candidates to be tested for their anti-viral potential. Bojsen et al. (2007) reported the antiviral effect of bovine whey proteins, whereas Civra et al. (2015) very recently identified from donkey milk lactadherin a peptide displaying anti-Rotavirus activity. The actual role of LAB proteolysis in the decrypting of the antiviral potential is far to be fully elucidated. An overview of antibacterial peptides is proposed in **Table 1**.

**Table 1:** Main antibacterial peptides from LAB.

Bacteria	Peptide	Protein source	Target	Author
LAB	Isracidin	α s1-Casein	*S. aureus*	Meisel and Bockelmann, 1999
LAB	k-Casedicin	k-Casein	*S. aureus*	McCann et al., 2006
LAB	Kappacin	k-Casein	*S. mutans*	Malkoski et al., 2001
LAB	Casocidin I	α s2-Casein	*E. coli* *S. carnosus*	Zucht et al., 1995
*L. helveticus*	Isracidin-like	β-Casein	*S. aureus* *L. innocua* *Yersinia* *Salmonella*	Minervini et al., 2003
*L acidophilus* *L. helveticus* *L. plantarum* *L. rhamnosus*	ND	In toto casein	*Entobacteria sakazakii*	Hayes et al., 2006
ND	Lactoferricin	Cow lactoferrin	*K. pneumoniae* *S. aureus* *S. mutans* *E. coli ol57*	Luo et al., 2007; Muro-Urista et al., 2011
*L lactis* *L bulgaricus* *L delbrueckii* *S. thermophilus*	ND	Goat casein	*S. aureus* *L. monocytogenes* *L. innocua* *Salmonella* *Enterobacteria*	Atanasova et al., 2014
*L lactis* *L bulgaricus* *L delbrueckii* *S. thermophilus*	ND	β-lactoglobulin	*S. aureus* *L. monocytogenes* *L. innocua* *Salmonella* *Enterobacteria*	Atanasova et al., 2014

*ND, not determined.*

### Metal-Binding Peptides

Some LAB strains digest casein, releasing casein phosphopeptides (CPP) phosphorylated on Ser residues. CPP have a strong anionic character and hence are very resistant to further proteolytic degradation (Sharma et al., 2011). They have been identified during cheese ripening due to microbial protease activity (Singh et al., 1997). The typical cluster is Ser(P)-Ser(P)Ser(P)-Glu-Glu, but they may contain also phosphorylated cysteines. Even if this motif is crucial for metal-binding, various phosphopeptide fractions revealed significant differences in this capability, which may be due to variant amino acid composition around the phosphorylated region. Hence, other factors can affect CPP-metal interaction, namely the total number of amino acids and the total negative charge (Cross et al., 2005).

It has long been established (Gagnaire et al., 1996) that beta and k-caseins (being poor in hydroxylated amino acids and therefore less suitable for phosphorylation) generate peptides less active in metal binding, whereas the Ser/Thr rich alpha s1 and alpha s2 caseins are the most suitable for chelating cations by means of CPP. However, alpha s1 casein-derived peptide (59–79), although bearing five phosphate groups, is less active in allowing mineral uptake by the human tumor cells HT29 than beta casein-derived peptides (1–25). Actually, besides the typical cluster sequence, specific secondary structure motifs in the bioactive peptide (such as beta-turn and loop), as well as the degree of aggregation of CPP in presence of divalent cations, are required for a correct mineral absorption (Ferraretto et al., 2003).

Casein phosphopeptides can chelate up to 250 mgCa/g with a dissociation constant in the mM range, with an affinity even higher for trace elements like zinc, iron, and copper (FitzGerald, 1998). Their mechanism of action rely on the fact that they can form soluble complexes with calcium even at alkaline pH (Berrocal et al., 1989). This results in increased absorption of calcium in the intestine, useful in the treatment of osteoporosis. Re-calcification of dental enamel and prevention of dental caries were also reported (Reynolds et al., 2008). Enhanced gut bioavailability and potential higher absorption of enzyme co-factors such as iron, zinc, copper, selenium, magnesium, and manganese, due to CPP was also described (Meisel and Olieman, 1998). On the other hand, controversial clinical results concerning both calcium (Korhonen and Pihlanto, 2006) zinc (Miquel and Farré, 2007) and calcium and iron absorption obtained on patients receiving CPP supplementation and controls, underline the difficulty to standardize the different supplements (Teucher et al., 2006). Finally, calcium availability is strongly dependent on meal composition (i.e., phytates) and complex interactions between different foods ingested.

In spite of all these considerations, all described peptides may constitute interesting tools for microelements nutritional improvement. In our aging western society bone disease is the third cause of nursery care and osteoporosis is not fully controlled by a healthy lifestyle (active movement and diet; Wei et al., 2003). Therefore, calcium-enriching supplements such as CPP open new nutraceutical perspectives. However, the need to use purified and well-characterized CPP (for having a reproducible nutritional impact) highly affects the costs. Hence, the use of LAB as probiotics or food supplements to obtain functional food, especially milk based drinks and yogurts will be a promising strategy. *Lactobacillus helveticus* LA, is a LAB adapted to the cheese environment displaying good proteolytic activity. It can decrypt from alpha casein a peptide showing calcium-binding activity (Dimitrov, 2009). *Lactobacillus helveticus* LBK16H can decrypt the tripeptides Ile-Pro-Pro and Val-Pro-Pro, both exerting anabolic effect on bones (Griffiths and Tellez, 2013).

### Antioxidant Peptides

The existence of antioxidant peptides in vegetal food (especially cereals and legumes) is a well-established concept, recently reviewed by Malaguti et al. (2014). Marine food, such as algae (Sheih et al., 2009) and squid (Rajapakse et al., 2005b), as well as unusual proteins like royal jelly-derived (Guo et al., 2009), yak milk casein (Mao et al., 2011), goat milk casein (Li et al., 2013), donkey milk (Piovesana et al., 2015), but also egg white lysozyme (Rao et al., 2012), can be a source of antioxidant peptides. Bacteria have the capability to decrypt antioxidant peptides from several food proteins. Antioxidant activities were described in fermented mussel sauce (Rajapakse et al., 2005a), in LAB-fermented milk whey (Virtanen et al., 2007), in fermented shrimps (Faithong et al., 2010), during sourdough fermentation of wheat, rye, and kamut flours (Coda et al., 2012) and in peptides derived from bovine casein after 24 h hydrolysis with *Bifidobacterium longum* (Chang et al., 2013). Antioxidant efficacy was also found in cell-free extracts of *Lactobacillus plantarum* isolated from traditional Chinese food (tofu and kefir) but it is rather related to pseudo catalase enzymes, exopolysaccharides and lipoteichoic acids than to bioactive peptides (Li et al., 2012). Recently, mixed cultures of LAB and yeast proved to be able to produce antioxidant peptides from cow milk, however, the peptide sequences having activity were not described (Li et al., 2015).

Antioxidant peptides can control both enzymatic and non-enzymatic peroxidation of fatty acids thus preventing membrane lipid peroxidation (Hartmann and Meisel, 2007). They can act as direct radical scavengers, non-radical oxygen quenchers or metal chelators, but they also can eliminate radical precursors, hence controlling ROS damage to cells and tissues, resulting in a beneficial action on the whole organism. The redox balance between ROS and antioxidant endogenous defenses is highly impaired during chronic inflammation (Zhang et al., 2015) thus, antioxidants are essential in controlling age-related chronic degenerative diseases concerning both the cardiovascular and the central nervous system.

Natural antioxidant peptides such as GSH (Cys-Glu-Gly) or carnosine (beta-Ala-His) play important roles in radical scavenging (Power et al., 2013). Antioxidant properties have been demonstrated for Tyr, Trp, His, and Lys, in particular when Tyr and Trp are in the C-terminus and the hydrophobic residues Val and Leu at the N-terminus of a peptide. Trp and Tyr behave as antioxidant molecules since their indolic and phenolic groups can act as hydrogen donors (Hernández-Ledesma et al., 2008). Basic amino acids can chelate metallic ions and cysteine acts as a proton donor, thanks to its thiolic group but the highest antioxidant activity was demonstrated for the tripeptide Pro, His, His (Malaguti et al., 2014). Furthermore it was demonstrated that Leu-His-His and Pro-His-His can both act synergically with non-peptide antioxidants such as butylated hydroxyanisole (Kitts and Weiler, 2003).

### Immunomodulatory Peptides

It is well established that some milk proteins (caseins, whey proteins, lactoferrin, lactoperoxidase) are able to control lymphocyte proliferation (Möller et al., 2008). Milk-derived peptides such as the C-terminus sequence of beta casein (193–209) can increase proliferation of rat lymphocytes (Meisel and Bockelmann, 1999), whereas alpha casein C-terminal hexapeptide (194–199) and beta casein fragments (63–68 and 91–93) induce macrophage maturation and phagocytosis enhancement *in vitro* (Fiat and Jollès, 1989). In agreement with these data, studies *in vivo* show that alpha casein-derived peptides can stimulate macrophages phagocytosis exerting a protective effect against *Klebsiella pneumoniae* infection in mice (Migliore-Samour et al., 1989). Short-chain peptides from milk whey proteins also stimulate the proliferation of murine spleen lymphocytes *in vitro* (Mercier et al., 2004). More detailed studies report that proliferation of human peripheral blood lymphocytes is induced by the k-casein and alpha lactalbumin fragment Tyr-Gly-Gly (Kayser and Meisel, 1996; Sharma et al., 2011). Natural killer (NK) cell activity and antibody synthesis can also be enhanced by food–derived bioactive peptides (Hartmann and Meisel, 2007). However, milk-derived peptides do not act only as simply stimulators of the immune system: immune-modulating activities including cytokines regulation and attenuation of allergic reactions has also been described (Korhonen and Pihlanto, 2003; FitzGerald and Murray, 2006).

Transferring clinical data to food is not so simple. Actually, in this scenario, LAB (especially probiotic LAB) play a very central role: beta-casein medium fermented with LAB gives rise to bioactive peptides acting on monocytes, macrophages and T helper cells, particularly with Th1-like cells (Laffineur et al., 1996). *Lactobacillus paracasei* hydrolyzes beta-lactoglobulin originating peptides stimulating Interleukin 10 (IL-10) production and downregulation of IL-4 and gamma interferon secretion (Prioult et al., 2004). Casein hydrolysated by *Lactobacillus rhamnosus GG* displays modulating effects (both stimulation and suppression) on lymphocyte proliferation (Möller et al., 2008). Furthermore, casein hydrolysates from *Lactobacillus rhamnosus GG* can induce enhancement of anti-inflammatory cytokines from Th1 lymphocytes and a parallel decrease of pro-inflammatory cytokines and immunoglobulins produced by Th2 lymphocytes, thus controlling allergic reactions (Delcenserie et al., 2008). *Lactobacillus helveticus* proteolysis products down-regulate cytokines production and stimulate macrophage phagocytosis (Matar et al., 2001) and also show protective effects against *E. coli O157* (LeBlanc et al., 2004) and *Salmonella typhimurium* (Tellez et al., 2011) infection. All the non-proteolytic strains of *Lactobacillus helveticus* failed to trigger immune system modulation. Three immune-active peptides derived both from beta casein and from alpha lactalbumin after milk fermentation with proteolytic strains of *Lactobacillus helveticus* were analyzed: they contain high percentage of proline histidine and lysine (Tellez et al., 2010). Establishing how, when and in what extent proteolysis occurs in food (and in the gut) is still an open question.

### Cell Cycle and Apoptosis Modulating Peptides

Milk-derived peptides, sometimes resulting from microbial proteolytic activity (Roy et al., 1999), can regulate cell growth, differentiation, and apoptosis (Roy et al., 2002). De Simone et al. (2008) demonstrated that waste milk whey is able to induce apoptosis in CaCo2 cancer cells, but also interfere with the cell cycle (reducing the number of cells in S-phase and enhancing the number of cells in G1 phase), thus inhibiting proliferation. Cooked milk whey loose this property probably due to irreversible denaturation of proteases decrypting the peptides of interest. The endogenous microflora of milk (both starters and contaminating bacteria) seems to be the main responsible of the phenomenon observed, since fresh milk does not exhibit this behavior (De Simone et al., 2008). Although the involved peptides were not characterized, it is very well known that some sequences such as Arg-Gly-Asp-Asp-Asp-Asp-Asp-Asp-Asp-Asp-Asp can have anti-proliferative effects on cancer cells models: the triplet Arg-Gly-Asp can account for adhesion to the extracellular matrix whereas the eight Asp residues can act intracellularly by binding chromatin (Galvez and de Lumen, 1999). Meisel and FitzGerald (2003) also demonstrated that normal cells are less sensitive to the apoptotic induction than malignant cells. Tumor cells apoptosis was also detected in an *in vivo* model in which mice were treated with milk that underwent fermentation by a highly proteolytic strain of *Lactobacillus helveticus.* On the contrary, the use of low-proteolytic strains did not gave satisfactory results in terms of breast cancer control in the same mice (De LeBlanc et al., 2005). On the other hand, not all anticancer effects of milk are related to proteolysis. It has been demonstrated that alpha lactalbumin can form a complex with oleic acid in acid conditions. This modified protein can induce apoptosis in cancer cells *in vitro* (Svensson et al., 2000). Therefore, is it tempting to hypothesize that acidification caused by LAB metabolism can also contribute to these effects, and further experiments proving this will be crucial to confirm such hypothesis and opening new perspectives for cancer control. Taken together all these results suggest that it is possible to increase host defenses against tumor degeneration by using food fermented with LAB.

### Opioid and Anti-opioid Peptides

These molecules constitute a promising frontier for treating both stress-related behaviors such as anxiety and depression and season-related mood disorders. They act on the appetite/satiety as well, as demonstrated by Pfluger et al. (2012) who named these molecules “nutropioids.” In general, these peptides are able to control the gut–brain axis at several levels, including gut–brain communication, brain cognitive function, and behavior (Cryan and Dinan, 2012). Their mechanism of action includes stimulation of receptors (K, delta, and mu) present in both the central and the peripheral nervous system and subsequent inhibition on adenylate cyclase activity. Some of these compounds are summarized in **Table 2**.

**Table 2:** Overview of the opioid and anti-opioid peptides.

Peptide	Protein source	Effect	Receptor	Author
β-Casomorphin	β-Casein	Opioid	μ	Loukas et al., 1983
ND	α S1-Casein	Opioid	δ	Loukas et al., 1983
Casoxin	k-Casein	Anti-opioid	μk	Chiba and Yoshikawa, 1986
Exorphin B5I	Gluten	Opioid	Out of the BBB	Fukudome and Yoshikawa, 1992
Soymorphine	Soy	Opioid	μ	Kaneko et al., 2010
Rubiscolin	Rubisco	Opioid	δ	Yang et al., 2001

The best studied myorelaxant peptides are beta-casomorphins (which act on the mu receptors) and alpha s1 casein-derived peptide (which acts on the delta receptor). Their primary sequences consist of 4–10 amino acids whose N-terminal residue, (essential to trigger biological activity), is Arg in alpha s1 casein-derived peptide and Tyr in beta-casomorphins (Loukas et al., 1983). This difference, together with the presence of six proline residues in beta-casomorphins, can account for the higher resistance to enzymatic digestion of the latter, resulting in enhanced half-life and absorption in human digestive tract. Once in blood, beta-casomorphins can reach receptors in the brain and in the peripheral tissues thus exerting a relaxing action inducing calmness and sleeping (Chabance et al., 1998). Another interesting peptide derived from alpha s1casein is alpha-casozepine, displaying anxiolytic action but not directly interacting with the mu and delta receptors. It has been demonstrated that the biological effect is mediated by activation of serotonin and GABA_A_ receptors, causing release of endogenous serotonin, dopamine, and GABA (Mizushige et al., 2013). Conversely, casoxins peptides (originated from k-casein hydrolysis) behave as opioid-antagonists over both the mu and k-type receptors. Due to this different physiological role, casoxins can be employed to counteract depression (Chiba and Yoshikawa, 1986).

Peptides produced with the contribution of LAB protease system on dairy proteins are named exorphins and casoxins, the former having opioid–like and the latter opioid-antagonist function (Chabance et al., 1998). However, other foods may contain opioid/anti-opioid peptides. It has long been established that wheat gluten is a good source of opioid peptides (Fukudome and Yoshikawa, 1992), and more recently, it has been shown that gluten exorphin B5 can enhance prolactine secretion by acting on receptors present outside the blood brain barrier. In this case, the biological effect is linked to a reduced dopaminergic tone (Fanciulli et al., 2005). Soymorphins are very interesting opioid peptides acting on mu-receptors present in the gut and connected with the serotonin, dopamine, and GABA receptors systems. It was shown that soy-derived opioid peptides act as anorexigenics and also reduce gastrointestinal motility (Kaneko et al., 2010). A delta-opioid peptide called rubiscolin was decrypted from the large subunit of spinach rubisco (Yang et al., 2001). The rubiscolin amino acid sequence is Tyr-Pro-Leu-Asp-Leu-Phe and it displays an analgesic effect besides being involved in memory consolidation (Yang S. et al., 2003). Hirata et al. (2007) also report anxiolytic action of this natural peptide by activation of the sigma1 and D1 dopamine receptors, when delivered orally (100 mg/Kg). The replacement of Leu^3^ with Ile or Met enhances by a factor four the opioid activity, whereas the replacement of Phe with Val potentiates the opioid activity more than 10-fold. The overall activity of the sequence Tyr-Pro-Met-Asp-Leu-Val is 20 times higher than the natural rubisco derivative (Yang S. et al., 2003).

### Antithrombotic Peptides

Blood coagulation occurs through a complex system of proteins undergoing a sequential proteolytic cascade. These proteins include pro-enzymes such as fibrinogen that is proteolytically activated to fibrin by means of thrombin. Bovine k-casein derived oligopeptides (fragment 106–116, 11 amino acids long) named casoplatelins (or thrombin inhibitory peptides), can inhibit the binding of human fibrinogen (gamma chain) on the platelet surface receptor, due to sequence homology between fibrinogen gamma chain and K-casein. Actually, the mechanism involved in milk clotting, defined by interaction of k-casein with chymosin bear a remarkable similarity to the process involved in blood clotting, defined by interaction of fibrinogen with thrombin (Qian et al., 1995). As a result, they prevent aggregation of ADP-activated platelets. Also the glycopeptide present on the N-terminus of k-casein, a smaller molecule called casopiastrin, displays inhibiting activity on fibrinogen binding on the platelet membrane (Ricci-Cabello et al., 2012). Although these molecules are mainly the result of human proteolysis on milk, LAB proteolytic activity on casein can contribute to their release both in food and *in vivo*. Casoplatelins are present in high amounts in the gut but, after absorption, significant concentrations are still bio-available in blood thus contributing to thrombosis control in humans.

### Antihypertensive Peptides

*Lactobacillus helveticus, Lactobacillus delbrueckii* subsp. *Bulgaricus* SS1, *Lactobacillus delbrueckii* subsp. *lactis*, and *Lactococcus lactis* subsp. *cremoris* FT4 can modulate blood pressure by producing angiotensin 1-converting enzyme inhibitory peptides (ACE inhibitors) from milk proteins. ACE is a carboxypeptidase converting angiotensin I (a decapeptide) into angiotensin II (an octapeptide) having a strong vasoconstrictor action (Hartmann and Meisel, 2007). *Lactobacillus delbrueckii* subsp. *lactis* hydrolyzes both alpha s1 and beta casein (but not k-casein) by means of a cell envelope proteinase releasing antihypertensive peptides: the proteolytic activity was maximal during the logarithmic growth and addition of NaCl and glycerol prevents correct proteolysis (Hébert et al., 2008). Casein-derived anti-hypertensive peptides are 2–6 amino acids long oligopeptides. Generally, LAB peptidases, by shortening the poly/oligopeptide chain, contribute to enhance the anti ACE potential. Actually the 6 (alpha s1 casokinin-6 = Thre-Thre-Met-Pro-Leu-Trp) and the 5 (alpha s1 casokinin-5 = Phe-Phe-Val-Ala-Pro and betacasokinin = Lys-Val-Leu-Pro-Val) amino acids long oligopeptides are less active than the tripeptides made up of Val-Pro-Pro and Ile-Pro-Pro. A very small dipeptide Tyr-Pro, proved to be effective in blood pressure control as well (Yamamoto et al., 1999). The final active short peptides are resistant to both pH variations and human digestive enzymes thus opening interesting applicative perspectives (Gobbetti et al., 2004).

### Cholesterol-Lowering Peptides

Hypocholesterolemic peptides can be originated by beta-lactoglobulin, casein and soy proteins proteolysis (Hori et al., 2001). They can lower the total cholesterol of rats *in vivo.* Their mechanism of action probably consists in the reduction of the micellar cholesterol solubility or also to an enhanced ability to bind taurocholate (Nagaoka et al., 2002). They overall action prevents cholesterol absorption by CaCo2 cells *in vitro* and enhances fecal steroid excretion *in vivo*. The sequences displaying such activities are Ile-Ile-Ala-Glu-Lys, Ala-Leu-Pro-Met-His and Gly-Leu-Asp-Ile-Gln-Lys (Nagaoka et al., 2001).

### Glucose-Uptake Stimulating Peptides

It has been demonstrated that dipeptides containing branched chain amino acids (Ile-Ile, Leu Leu, Ile-Leu, Leu-Ile, Ile-Val, Leu-Val, Val-Leu) can enhance glucose uptake in skeletal muscle cells, favoring glycogen synthesis and thus controlling hyperglycemia (Morifuji et al., 2010). Generally, glucose uptake by skeletal muscle cells is induced by exercize-training by means of increased expression or activity of key-signaling proteins (Goodyear and Kahn, 1998). Branched-chain amino acids are the main nitrogen source for skeletal muscles and milk whey proteins are particularly rich in branched chain amino acids (22.3%) as compared to caseins (20.3%), soy proteins (17.5%), or wheat gluten (14.1%; Morifuji et al., 2010). The dipeptide Ile-Leu is the most abundant after milk whey hydrolysis. Therefore, assessing the proteolytic activity of LAB toward milk whey proteins (that constitute natural nitrogen substrates for LAB), is a promising strategy to get nutritional supplements improving health. Proteomic approaches using LC-MS-MS can be a valuable help for identifying small nutritionally relevant peptides and a new discipline, called food peptidomics, is now expanding (Lahrichi et al., 2013).

### Chaperone-Like Peptides

A very interesting class of protein-encrypted peptides are those displaying chaperone-mimetic action (Bumagina et al., 2009; Artemova et al., 2010). Synthetic peptides were constructed on the model of bovine hemorphin-6, wheat gluten exorphin C, and spinach rubiscolin-5 (all opioid peptides), and their ability in refolding model proteins was investigated. The target proteins to be refolded were heat-treated or DTT-aggregated carbonic anhydrase, ADH and bovine milk alpha lactoglobulin. The results clearly indicate that the peptides are able to refold damaged proteins thus opening the way to the interesting hypothesis that food proteins, besides their nutritional role, can act as systems involved in quality control of proteins especially during stress (Artemova et al., 2010).

### Mixed Function Peptides

Some food encrypted peptides, in case decrypted by LAB proteolytic action, possess mixed function. In **Table 3** are reported the peptides displaying two or more biological activities.

**Table 3:** Overview of peptides with mixed functions.

		Activity					
Peptide	Protein source	Immuno-modulator	Cell cycle	Anti-ACE	Opioid	Anti-microbial	Author
β-Casomorphin-7 (YPFPGPI)	β-CN	+	+		+		Hatzoglou et al., 1996a,b
β-Casokinins-10 (YQQPVLGPVR)	β-CN	+		+			Hartmann and Meisel, 2007
α-Lactorphin (YGLF)	α-LA			+	+		Meisel and Bockelmann, 1999
β-Lactorphin (YLLF)	β-LG			+	+		Meisel and Bockelmann, 1999
Antimicrobial pept ide (AGTWY)	β-LG	+				+	Hernández-Ledesma et al., 2008
Immunopeptide (YGG)	α-LA	+	+			+	Sharma et al., 2011

As an example the tripeptides Val-Pro-Pro and Ile-Pro-Pro, released from beta and K-casein by *Lactobacillus helveticus* are immunomodulatory and hypotensive (Hartmann and Meisel, 2007). This effect can be explained by the fact that ACE inhibition favors bradykinin production, and bradykinin plays a crucial role in the inflammation process by stimulating macrophages and increasing lymphokines secretion by lymphocytes (Sharma et al., 2011).

Some immunostimulatory peptides also exhibit antimicrobial features: beta-lactoglobulin-derived peptides can improve phagocytosis but also stimulate the microbial autolytic system. This is not only restricted to bacteria but also to fungi and both naturally autolyzing and non-autolyzing strains are sensitive to this effect (Hernández-Ledesma et al., 2008).

It has long been established that opioid peptides such as casomorphin agonists also decrease cell proliferation by acting on somatostatin receptors (Hatzoglou et al., 1996a,b). Since opioid and somatostatin receptors are present on different cells including central nervous, endocrine and immune system (De Simone et al., 2008) the action of such peptides is more complex than expected.

Several peptides are opioid and hypotensive, especially those derived from bovine milk proteins (casomorphins, lactorphins) and beta-casomorphin 7 is opioid, hypotensive, and immunomodulatory: this last function is targeted on lymphocyte proliferation that is sometimes stimulated and sometimes inhibited depending on the peptide concentration (Meisel and Bockelmann, 1999).

Lactoferricin, the lactoferrin-derived antimicrobial peptide, besides being an antibacterial, antifungal and antiviral molecule, can also control carcinogenesis by means of its anti-inflammatory and immune-modulating properties. This effect seems to be due to a positively charged region of the peptide that is very rich in tryptophan and arginine residues (Sharma et al., 2011)

The reason of these mixed functions lies in the fact that some regions in the primary structure of caseins contain overlapping peptide sequences, exerting different physiological functions (Meisel and Bockelmann, 1999). These domains, highly hydrophobic and rich in proline residues, have been defined as “strategic zones” and are resistant to proteolytic attack (Fiat and Jollès, 1989).

In conclusion, of this paragraph it is important to underline that not all bioactive peptides released by the combined action of LAB proteolytic system and digestive proteases in food and in the gut can be available for humans. Actually, bioactive peptides may be degraded during digestion, may be poorly absorbed and hence reach the target tissues at a concentration lower than the one necessary to exert their biological function. As far as degradation is concerned, supplying bioactive peptides by means of GRAS bacteria (like LAB are) plus protein complements is simpler than encapsulate them and this constitutes therefore a promising strategy to transport the molecules to the intestine, preventing degradation in the upper digestive tract. Efforts have been made also to assess the real *in vivo* absorption of bioactive peptides: blood levels of antihypertensive peptides have been quantified (by LC ESI triple) after ingestion by human volunteers (van Platerink et al., 2006) but also *in vitro* studies measuring epithelial translocation across CaCo2 monolayers by means of ESI-LC-MS-MS can supply reasonable information about absorption. One challenging aspect of these approaches is the need of improved “omics” methods aimed to identify low- molecular weight bioactive peptides. MALDI (matrix-assisted laser desorption ionization) TOF-MS has proved to be inadequate because of matrix interference (Saavedra et al., 2013) and matrix-free methods such as NALDI (nanostructure-assisted-laser desorption ionization; Kütt et al., 2011) have been re-proposed.

## Bioactive Amines

A number of microorganisms synthesize, as decarboxylation products of precursor amino acids, biogenic amines. In LAB, expression (by transcriptional induction) and/or activation (by catalytic modulation) of amino acid decarboxylation systems are non-essential adaptive responses to energy depletion but also strategies to counteract acid stress (Pessione, 2012).

However, not all biogenic amines are bioactive molecules since most of them only act as spoilage compounds (putrescine, cadaverine) sometimes enhancing the toxicity of true bioactive molecules (Medina et al., 2003). On the contrary, the decarboxylation of certain amino acids, as tyrosine and histidine, can give rise to bioactive molecules (tyramine and histamine) involved in several pathogenic syndromes, more extensively described in the following paragraphs. On the other hand, not all bioactive compounds negatively affect human health: as an example, beta-phenylethylamine derived from phenylalanine or tryptamine originated from tryptophan can exert beneficial actions (such as mood control and appetite/satiety balance regulation) when administered to humans (Shimazu and Miklya, 2004). Similarly, it has been reported that several LAB (*L. bulgaricus, L. acidophilus, L. casei, L. plantarum*) can synthesize melatonin, a bioactive molecule, deriving from tryptophan decarboxylation and serotonin metabolism, acting on sleep and reproductive behavior but also controlling immunity, inflammation and carcinogenesis (Tan et al., 2014). A particular case is the glutamate product gamma amino butyrate (GABA): this molecule can be naturally present in food but also artificially added to enhance the nutraceutical value of a certain food (GABA-tea, GABA-rice; Abe et al., 1995; Oh, 2003) due to its relaxing action on muscles and to its overall beneficial effects on the nervous system.

### Bioamines in Food

Cheese is the food most frequently associated with a too high biogenic amine content. It is very rich in free amino acids due to the desired proteolytic action performed by both bacteria and fungi during “maturation,” when colonized by decarboxylase-positive LAB (either autochthonous or contaminant bacteria) it can be a source of tyramine, histamine, putrescine, and beta-phenylethylamine (Pessione et al., 2009). Tyramine and beta phenylethylamine are particularly abundant in food from animal origin (including fermented sausages) since these matrices are very rich in the precursor amino acids, tyrosine, and phenylalanine, used by animals to synthesize catecholamines. Both tyramine and beta-phenylethylamine are bioactive compounds whose action will be better described in the following paragraphs.

As far as fermented meat products are concerned, tyramine, cadaverine, putrescine, and histamine can be found (Singh et al., 2012). Poor quality processing favoring contamination is the main cause of a too high bio-amine content of meat, however starter strains possessing the capability to form biogenic amines, like *Lactobacillus curvatus*, have been described as well (Singh et al., 2012). To prevent such a risk, selection of particular starter cultures possessing either amino oxidase activity (Alvarez and Moreno-Arribas, 2014) or bacteriocin synthesis capability (Somda et al., 2011) should be taken into concern to contain the undesired consequences of spontaneous fermentations.

**FIGURE 2:**
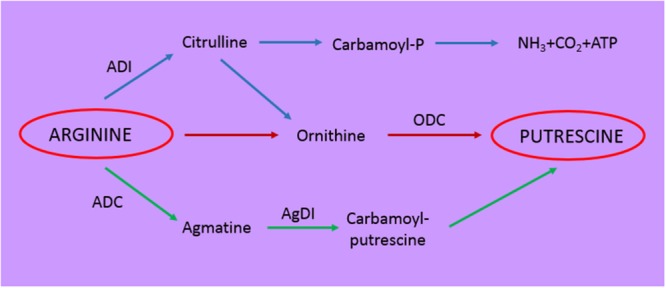
**Schematic representation of the metabolic pathways involved in putrescine formation.** ADI, arginine deiminase; ADC, arginine decarboxylase; AgDI, agmatine deiminase; ODC, ornithine decarboxylase.

Other fermented foods are alcoholic beverages such as beer, ciders, and wine, that being of vegetal origin, are generally poor of aromatic amino acid precursors (Lehtonen, 1996). At the end of alcoholic fermentation the only amino acid present in wine in significant amount is arginine (2,4 g/L): this can be converted by the arginine decarboxylase into agmatine and then putrescine, or rather be directed to the arginine deiminase pathway (ADI) generating ammonia, ornithine (generating putrescine) and carbamoyl phosphate (**Figure 2**) (Costantini et al., 2013). Both putrescine and ornithine are not true bioactive compounds even if they can alter the organoleptic properties of wine and enhance the toxicity of other biogenic amines (Costantini et al., 2013). Carbamoyl phosphate can combine with ethanol in wine generating ethyl carbamate a carcinogenic molecule (Jiao et al., 2014).

However, if the oenological procedures are not correctly performed, other bioactive amines can be generated. Actually, at the end of the alcoholic fermentation when all the grape sugars are exhausted, yeast cells die and undergo autolysis, releasing their own proteins and proteases (from lysosomes). Hence, if dead yeasts cells are not immediately separated from wine, free amino acids (from yeast proteins) become available for autochthonous LAB able to perform malolactic fermentation (MLF; Coton et al., 1999). This secondary fermentation is widely appreciated for its improvement of wine flavor, and it is usually performed by the predominant species *Oenococcus oeni* or, more occasionally, by other LAB species belonging to the genera *Lactobacillus* and *Pediococcus*, which may develop during the winemaking process (Lonvaud-Funel, 1999). Unfortunately, some LAB, besides TPP-dependent-acid decarboxylases, also bear PLP-or pyruvoil-dependent amino acid decarboxylases that can generate undesired amines, depending on the precursor amino acid present at this fermentation stage (Recsei and Snell, 1985). Therefore, histamine, cadaverine, tyramine, and beta-phenylethylamine (whose action will be better described in the following paragraphs) can be produced.

The total content of biogenic amines in food varies from 1 to 50 mg/Kg (alcoholic and no-alcoholic beverages, soy products) to 20–1,500 mg/Kg [sausages, cheeses; EFSA Panel on Biological Hazards (BIOHAZ), 2011] At high concentrations, BA are risk factors for food intoxication, while moderate levels may lead to food intolerance (Ladero et al., 2010) It is very difficult to establish a uniform maximum limit for ingested BA since their toxic effect depends on the type of BA in question. The unique BA for which the European Food Safety Authority (EFSA) has legally set maximum limits is histamine and only related to fish and fish products (Linares et al., 2016). Furthermore, the toxic effects exerted by amines possessing biological activity in humans, strongly depend on individual sensitivity to them (Santos, 1996). As an example, tyramine tolerance is lower in patients treated with antidepressant mono-amino-oxidase inhibitory drugs (IMAO), which interact with amino-oxidase enzymatic systems (MAO, DAO), since these patients have an impaired ability to oxidize amines. For the same reason, the toxic effects of bioactive amines in wine consumers is enhanced by the presence of ethanol, which is one of the most effective inhibitors of amine oxidases (Sessa et al., 1984).

### Factors Affecting Amines Production in LAB

When considering the risk of bioactive amine release into food, it is important to underline that maximum energy depletion and acidification occurs during the stationary growth phase. If (theoretically) LAB can be removed from food before resting, no amine accumulation takes place. This statement is supported by proteomic investigations that revealed that, in *L. hilgardii*, histamine production is induced only during stationary phase (Pessione et al., 2005). On the contrary, northern blotting experiments showed activation of the HDC (histidine decarboxylase) gene already during exponential growth phase in a different *L. hilgardii* strain (Landete et al., 2008). However, the same authors clearly demonstrated an inhibitory effect of both glucose and fructose (still high during exponential growth phase) over the genes coding for HDC in *L. hilgardii, O. oeni*, and *P. parvulus* (Landete et al., 2008). Furthermore, in both *L. hilgardii* and *L. brevis*, it has been demonstrated that tyramine accumulation is maximal in presence of either fructose or fructose plus L-malic acid (Moreno-Arribas et al., 2003) thus supporting the idea that it is not necessary to reach the stationary phase to trigger amine accumulation. Actually, also in a different bacterial model (*E. faecalis*) studies involving sub-proteomes profiling demonstrated a high tyramine content already in the exponential phase when sugars are not yet consumed (experiments performed in chemically defined medium having glucose as sugar carbon source; Pessione et al., 2009). Therefore, the evidences are still controversial and in part related to specific amino acid/amine reaction, in part linked to a strain difference.

A further strong evidence is the fact that amine production is related to the availability of the precursor amino acid. Supplements of tyrosine in the medium can increase about 10-fold tyramine accumulation and about twofold tyrosine decarboxylase (TDC) activity in *L. brevis* (Moreno-Arribas et al., 2000). Similarly, in *Enterococcus faecalis*, TDC was found to be more abundant in the membrane extracts when the strain was exposed to free tyrosine (Pessione et al., 2009). Histamine is biosynthesized only in presence of histidine: the cytosolic enzyme HDC was found more abundant in cells grown in histidine medium (stimulated condition) in *Lactobacillus hilgardii*, and *Lactobacillus 30a* (Pessione et al., 2005; Mazzoli et al., 2009) as compared to control conditions.

The only exception to this rule seems to be the GAD enzyme that can allow GABA accumulation even in the absence of glutamate. However, the chemically defined-medium medium (CDM) used for growing *L. lactis* contains glutamine, so it is possible that part of it is converted to glutamate that can undergo decarboxylation to GABA. On the other hand, a transcriptional control exists since addition of glutamate can trigger a light enhance of GABA biosynthesis (Mazzoli et al., 2010).

A critical concern connected with the necessity of free amino acid availability is how bacteria can get them. As far as LAB are concerned, often in the same ecological niche where they live proteins are available (cheese) or microbial autolysis (wine) generates free proteins that can undergo proteolytic breakdown by LAB (Lamberti et al., 2011).

Interestingly, other factors can affect bioactive amine production and, among these, acids (that can undergo or not a similar decarboxylative process). It has been demonstrated that L-lactic acid does not influence both histamine accumulation and HDC biosynthesis (Landete et al., 2008). Similarly, malate has no effect on histamine accumulation (Mangani et al., 2005) or histidine consumption (Mazzoli et al., 2009). In agreement with these data, Moreno-Arribas et al. (2000) showed that tyramine production was not affected by either malate or citrate, whereas Lonvaud-Funel (2001) observed that both tyramine and histamine accumulation was lower during MLF and higher after it. The utilization of the amino acids tyrosine and histidine only after malate does not necessarily indicate malate inhibition, but simply that bacteria prefer utilize carbon sources before consuming nitrogen compounds.

Other factors affecting amine accumulation in food include temperature, spices and salt content, generally controlling microbial growth. The optimum growth for most amine-producing bacteria is 20–37°C. Below this temperature, the risk to find biogenic amines in food decreases (Karovičová and Kohajdová, 2005). The sausage diameter as well as the addition of spices can control a too high amine content in meat: the larger the size, the higher the risk of amines due to higher water activity and lower salt content. However, NaCl not only negatively affects bacterial growth, proteolytic activity and histidine decarboxylase activity but also stimulates both tyrosine and glutamate decarboxylase (GAD) synthesis acting as operon inducer (Singh et al., 2012).

### Histamine

The ingestion of histamine-rich food may cause heterogeneous symptoms such as diarrhea, arrhythmia, headache, and allergic syndromes such as respiratory distress (asthma), rhino-conjunctivitis, urticaria, pruritus, and flushing (Manzotti et al., 2016). Histamine is a strong hypotensive compound also acting on many receptors present in the gastro intestinal tract (Santos, 1996). Hence, high content of histamine is responsible of the toxicity of spoiled or not properly stored fish. In black carp fillets, for example, the contents of BAs remained invariable during the early stages of storage and began to increase at the later stages of storage occurs during food storage. In particular, a significantly higher concentration of histamine (132.05 mg/kg on the third day) was detected in the black carp filets stored at 20°C, indicating that this temperature favors the formation of histamine (Hongbing et al., 2016). In wines, especially red wines undergoing spontaneous MLF by LAB, are frequently responsible of these syndromes. Amino acids generating bioactive amines (histidine, tryptophan, glutamate) can be released during the, post-alcoholic fermentation, by yeasts autolysis as referred above (Alexandre et al., 2001). The concentration of free amino acids in wine is also dependent on the proteolytic activity of the strain concerned with MLF (Alexandre et al., 2001).

### Tyramine

Tyramine is a bioactive molecule acting both at vascular level (hypertension, vasal-constriction) and on the central nervous system (headache). Despite it is generally assumed that histamine is the most toxic BA, a recent work reveals that unexpectedly, tyramine is more cytotoxic than histamine on an *in vitro* model of the human intestinal epithelium. Furthermore, the concentrations found to be toxic are commonly reached in BA-rich foods (Linares et al., 2016). Severe syndromes have been described in patients lacking a correct amino-oxidase activity because in treatment with mono-amino-oxidase inhibitory drugs (MAOI). MAOI are used as antidepressant because they prevent the oxidative catabolism of brain-active amines, such as dopamine, serotonin, tryptamine, and β-phenylethylamine. Unfortunately, MAO-inhibitors cannot discriminate among the different MAOs, and their overall effect results in high concentrations of tyramine that can cause hypertensive crises, brain hemorrhage and sometimes death (Pessione et al., 2009). Since tyramine is the biogenic amine most frequently found in cheese (due to the referred high tyrosine content of dairy products) this syndrome is also called “cheese reaction.” For the reasons explained above, MAOI development has led to compounds with improved tolerability profiles compared to the initial irreversible MAOIs, particularly in regard to tyramine-induced hypertension and dietary restrictions (Yanez et al., 2012). The huge number of non-controlled autochthonous bacteria involved in cheese manufacturing and the accidental contamination by unwanted strains during storage render cheese a risky food for tyramine ingestion, although starters are accurately typed. TDC activity has been described in several LAB strains of the genera *Lactobacillus, Carnobacterium, and Leuconostoc.* The enzyme from *Lactobacillus brevis* and *Enterococcus faecalis* has been fully characterized. Proteomic studies reveal the membrane location of such a protein in *Enterococcus faecalis* (Pessione et al., 2009), thus confirming the high hydrophobic nature of this enzyme (previously predicted on the basis of gene analyses suggesting large hydrophobic domains but also because of the harsh procedures needed to purify it). Interestingly, *Enterococcus faecalis* TDC proved to be able to produce not only tyramine from tyrosine but also beta-phenylethylamine from phenylalanine (Pessione et al., 2009).

### Beta-Phenylethylamine

β-Phenylethylamine, the decarboxylation product of phenylalanine, behaves like an endogenous amphetamine. It can control appetite/satiety thus being useful to treat obesity and related syndromes as well as in weight-control diets. Like amphetamine it can also positively affect mood. However, controversial effects including insomnia, anxiety, and a MAO-blocking activity that may extend the half-life of tyramine thus potentiating its toxicity (Millichap and Yee, 2003) have been described. Evidence of a specific phenylalanine decarboxylase enzyme in bacteria, has been reported only for *Bacillus cohnii*, a *species* taxonomically distant from LAB. Nevertheless, both Lactobacilli and Enterococci, frequently found in fermented food, display a clear β-phenylethylamine accumulation. Originally, some authors (Millichap and Yee, 2003) suggested that the β-phenylethylamine found in fermented food is the result of the activity of tyrosine decarboxylating bacteria. It is interesting to underline that in mammals and in several eukaryotic cells models a low-selective aromatic L-amino acid decarboxylase (EC 4.1.1.28) exists. In bacteria, enzymatic studies proved that TDC from *E. faecalis* is active also toward DOPA, but not toward other amino acids like phenylalanine while *Lactobacillus* bears a highly tyrosine selective TDC. More recently, the presence in *E. faecalis* of phenylalanine-decarboxylating TDC (EC 4.1.1.25) has been clearly demonstrated by a bi-phasic kinetic of tyramine and β-phenylethylamine production (Pessione et al., 2009). Tyrosine is consumed first and it is fully converted into tyramine (during logarithmic growth). Then phenylalanine is decarboxylated to β-phenylethylamine, although with lower conversion rate and yield. These results are consistent with a double catalytic activity of the same protein (TDC) having higher specificity for tyrosine and only low affinity for phenylalanine. On the other hand, Marcobal et al. (2006) observed β-phenylethylamine accumulation in a recombinant *E. coli* expressing the TDC gene of *E. faecium*.

### GABA

γ-Aminobutyric acid is one of the bioactive amines having true positive effects on human health. It acts as a neurotransmitter in the central nervous system of most vertebrates and plays additional roles on the overall human physiology such as lowering the blood pressure in mild hypertensive patients and acting as smooth muscles relaxation system (Mazzoli et al., 2010). Bacteria can produce GABA by the decarboxylation of glutamate through GAD. Glutamate and GABA act as opposite molecules having excitatory and inhibitory roles, respectively (Hyland and Cryan, 2010). *Lactococcus lactis* NCDO 2118 can obtain GABA from glutamine opportunely converted into glutamate (Mazzoli et al., 2010). It has long been established that *L. lactis* possess only one gene encoding GAD and that this enzymes is highly specific for glutamate (apparent Km 0.51 mM). Its molecular weight is about 54 kD and it has maximal activity at pH 4.7, whereas no activity is present at neutral pH (Nomura et al., 1999). Differences have been reported between *Lactococcus lactis* subsp. *lactis* and *L. lactis* subsp. *cremoris*: both possess gadCB genes encoding for GAD, however, one-base deletion of adenine and one-base insertion of thymine were detected in the coding region of the latter rendering it unable to synthesize a functional GAD enzyme (Nomura et al., 2000). Lactobacilli are the best GABA-producers, however, Lactococci, Streptococci, and Bifidobacteria can synthesize GABA as well (Lyte, 2011). Several strategies have been proposed to obtain high GABA amounts from cheap substrates through LAB sourdough fermentation (Coda et al., 2012), batch fermentation using immobilized (Huang et al., 2007) or entrapped (Choi et al., 2006) *Lactobacillus brevis* cells for the construction of functional food. To achieve maximum GABA production, temperature and pH are crucial (Dhakal et al., 2012). Nowadays, several nutraceutical preparations, such as GABA tea (Abe et al., 1995), GABA rice (Oh, 2003), GABA soymilk (Tsai et al., 2006), and GABA chocolate (Nakamura et al., 2009) are available, especially in Asia. GABA natural content of cacao is about 0.009%, however, this value is lower when considering the market-available chocolate also containing milk, sugar, and other ingredients. Functionalized chocolate, containing 0.28% GABA, proved to be able to lower the level of salivary chromogranin A and cortisol, thus reducing psychological stress (Nakamura et al., 2009).

## Conclusion

The knowledge on how, when and where bioactive amines are produced by LAB present in fermented food will be helpful by one side to prevent food borne diseases but on the other side also to use food as a nutraceutical delivery system to control mood, appetite and smooth muscle relaxation.

The knowledge about food-encrypted peptides and their potential can offer, in the coming decades, new tools for valuable therapies for the treatment of infectious diseases as well as metabolic, nutritional and psychiatric disorders, cardiovascular diseases, allergies and cancer, as suggested by Lien and Lowman (2003). The increasing availability of mass spectrometry facilities together with homology-based identification of potential bioactive peptide domains on protein sequences allows *in silico* prediction of encrypted peptides and will in the future be essential for targeting the right proteins. Several data banks are available for the bio-informatics analysis of potential bioactive peptides (Iwaniak et al., 2005; Lahrichi et al., 2013), especially antibacterial peptides, due to the urgent issue to face antibiotic resistance (Khaldi, 2012; Mooney et al., 2013). APD2 and CAMP are databases set up to classify not only antibacterial but also antifungal and antiviral peptides (Wang et al., 2009; Thomas et al., 2010). An even larger target is supplied by BIOPEP, Pep Bank, and Peptide DB, also classifying peptide hormones, growth factors and cytokines (Shtatland et al., 2007).

A new term, “nutritional peptidomics” has been proposed by Panchaud et al. (2012) to underline the importance of characterizing (mainly by mass spectrometry) food-encrypted peptides displaying health effects. These approaches allow to set up procedures to liberate the hidden bioactive molecules by means of different proteolytic enzymes (pepsin, trypsin, chymotrypsin) but also to synthesize artificial peptides displaying bioactivity (Zambrowicz et al., 2013). Microbial fermentation is one of the most promising strategies to generate bioactive peptides, hence genomic and proteomic characterization of new strains to predict their proteolytic profile is a challenging approach in view of obtaining functional food. As far as LAB are concerned, combining the substrate specificity and the cleavage pattern of LAB proteases can highlight the strain potential to release health-promoting molecules (or possible dangerous compounds) in food (Hayes et al., 2007a,b). This is a valuable strategy for selecting the right starters and to enhance the number of LAB strains to be used as food additives or probiotics. Moreover, the use of industrial by-products, such as milk whey, as growth substrates can lower the cost of functional food production and furtherly improve nutraceutical food availability.

## Author Contributions

EP: Overall organization of the paper and content. SC: Figure and table management and critical reading.

## Conflict of Interest Statement

The authors declare that the research was conducted in the absence of any commercial or financial relationships that could be construed as a potential conflict of interest.
